# Pharmacist Assistants’ role in medicine supply management at a primary healthcare setting

**DOI:** 10.4102/hsag.v27i0.2041

**Published:** 2022-11-30

**Authors:** Sibusiso M. Zuma

**Affiliations:** 1 Department of Health Studies, College of Human Sciences, University of South Africa, Pretoria, South Africa

**Keywords:** distribution, medicine, post-basic pharmacist assistant, primary healthcare, medicine supply management

## Abstract

**Background:**

The need to improve essential medicine supply in the public sector is of paramount importance to ensure that the drugs used in treatment regimens are accessible, acceptable, safe, cost effective and affordable to the population.

**Aim:**

To assess the role of post-basic pharmacist assistants at primary healthcare clinics in order to make recommendations aimed at improving essential medicine supply management.

**Setting:**

The study covered five provinces namely: Eastern Cape, Free State, Gauteng, KwaZulu-Natal and Mpumalanga.

**Method:**

A qualitative, exploratory, descriptive research design was followed, using a semi-structured interview guide to collect data from 11 District Pharmaceutical Service Managers together with medicine availability reports review. Data analysis was performed using Colaizzi’s seven steps.

**Results:**

The study found that there is a shortage of post-basic pharmacist assistants in primary healthcare clinics, which affects the management and availability of medicine supplies. Placement of the post-basic pharmacist assistants would improve medicine supply management in the primary healthcare clinics.

**Conclusion:**

At least one post-basic pharmacist assistant should be appointed at each primary healthcare clinic in order to ensure efficient medicine supply management and consistent medicine availability.

**Contribution:**

The study provides evidence that primary healthcare clinics without post-basic pharmacist assistants are more likely to have erratic medicine supply management practices and confirms that post-basic pharmacist assistants play a positive role in the medicine supply management processes in primary healthcare clinics.

## Introduction

Essential medicine stockout is experienced in primary healthcare clinics, sometimes resulting in patients being turned away from facilities without receiving their prescribed medicines. At times, patients are advised by nurses or community healthcare workers to travel to other facilities presumed to have more stock (Hodes et al. 2017).

On average, member states of the World Health Organization (WHO) spend half of their total health expenditure on medical products. Improving access to essential medicine is a prerequisite for establishing universal health coverage and achieving the international goals relating to maternal and child health, as well as communicable diseases, specified in the Sustainable Development Goals (SDGs) adopted by the United Nations (WHO 2014). The need to improve the availability of essential medicines for chronic diseases, especially in the public sector, is of paramount importance to ensure that the drugs used in treatment regimens are accessible, acceptable, safe, cost effective and affordable to the population (WHO 2020).

According to the South African Pharmacy Council (SAPC) (2020), there is an appropriate workforce, (mid-level workers), registered in the category of post-basic pharmacist assistant with an approved scope of practice to supply medicine under the indirect personal supervision of a pharmacist at a primary healthcare clinic or any other facility in South Africa as approved by the SAPC.

In South Africa, out of the 3074 clinics surveyed in the Health Systems Trust National Healthcare Facilities Baseline Audit, 84% had no input from a pharmacist or post-basic pharmacist assistant (Health Systems Trust 2013). The implication of this finding is that the majority of service users of primary healthcare clinics receive essential medicines from nurses rather than from trained post-basic pharmacist assistants.

Mokgathla and Kadama (2017) reported that in the South African primary healthcare sector, medicine supply management is handled by nurses, who are required to divide their attention between patient care and medicine stock management and as a result, both functions are compromised.

Nurses are unable to cope with the additional function of medicine supply management because of the increased workload. In a study conducted in one province in South Africa, nurses reported a high workload at primary healthcare clinics where dispensing services had to be incorporated into an already time-constrained patient consultation schedule (Bobbins, Burton & Fogarty 2020).

Higher levels of medicine stockouts persist in the worst-impacted districts – and in many cases the same districts have suffered in this manner continuously year after year. It was recommended that the number of pharmacist assistants at primary healthcare clinics be prioritised, as these healthcare workers have the skills to deal with challenges related to the shortage in the supply of medicines (Stop Stockout 2018).

In response to the recommendation to improve compliance with the medicine standards, the South African government gazetted Human Resources for Health normative guides for clinics (National Department of Health 2015).

Empowered by the gazetted normative guides, provinces started to introduce the appointment of pharmacist assistants to take charge of the provision of essential medicines in the primary healthcare clinics. However, a number of provinces have been struggling to ensure that all primary healthcare clinics have at least one post-basic pharmacist assistant.

Although pharmacist assistants are deemed capacitated and skilled to play a key role in medicine supply chain management at the healthcare facilities, there is still a shortage of post-basic pharmacist assistants, leaving nurses to take responsibility for medicine supply chain management at most primary healthcare clinics (Matema 2020).

The limited availability of post-basic pharmacist assistants affects essential medicine supply management, as observed in the results of the National Core Standards assessment conducted in South Africa, which reflected that all primary healthcare clinics and community health centres were unable to meet the compliance requirements, including the provision of essential medicines and supplies (Matsoso, Fryatt & Andrews 2015). It is evident that, in the South African context, where the majority of the citizens can access health services at the primary healthcare clinics, the bulk of health services, including medicine provision, is offered by nurses – in the absence of post-basic pharmacist assistants.

### Problem statement

South Africa experiences medicine supply challenges in the nurse-run primary healthcare clinics. As an intervention, government introduced the appointment of pharmacist assistants to manage medicine supply in the primary healthcare clinics. Despite the efforts by the Department of Health to place pharmacist assistants in the primary healthcare clinics, medicine stockout continues to affect the primary healthcare service users. Limited studies have been conducted to investigate (post appointment) the difference that pharmacist assistants are making in medicine supply management within the primary healthcare clinics.

The study’s aim was to assess the role of pharmacist assistants at primary healthcare clinics in order to make recommendations for improving essential medicine supply management.

### Theoretical framework

Zuma and Modiba (2016) recommended that the medicine supply management framework, as developed by Management Sciences for Health (an organisation committed to essential medicine supply research) and adopted by the WHO, should be utilised as a guiding theoretical framework for essential medicine provisioning management.

**FIGURE 1:**
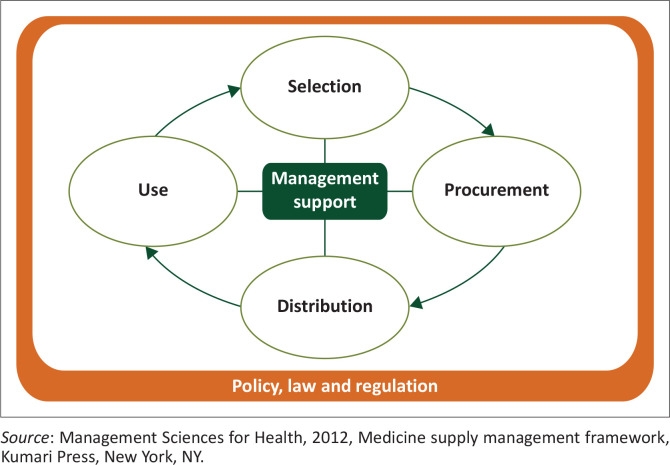
Medicine supply management framework.

The medicine supply management framework is focused on the selection, procurement, distribution and use of essential medicines, with related management support systems including planning and organisation of health services, financial management, information management and human resource management being central in the management of essential medicines (Management Sciences for Health 2012). The framework, as depicted in Figure 1, was used in the development of the research questions and interview guide, as well as reporting the findings of this study.

### Aim

The study’s aim was to assess the role of pharmacist assistants at primary healthcare clinics in order to make recommendations for improving essential medicine supply management.

### Research objectives

- To assess the medicine availability status in primary healthcare clinics with post-basic pharmacist assistants
- To determine the medicine supply management practices at primary healthcare clinics where there are post-basic pharmacist assistants
- To make recommendations to improve medicine supply management in the primary healthcare clinics.

## Research methodology and design

### Research design

A qualitative, exploratory research design was utilised to conduct this study. Data collection was executed through document analysis and semi-structured interviews with district pharmaceutical services managers identified as information-rich participants, as they have first-hand experience in the management of essential medicines. The data were analysed using the steps provided in Colaizzi’s (1978) data analysis approach (Creswell & Creswell 2018).

### Setting

The South African government provides primary healthcare services through health district platforms (Health Systems Trust 2013). In South Africa, there are 52 health districts spread across nine provinces, with the majority of them located in the rural areas. This study was conducted in 11 health districts having a total of 685 primary healthcare clinics, of which 429 had at least one post-basic pharmacist assistant, while 256 did not have a post-basic pharmacist assistant.

The study covered five provinces, namely Eastern Cape, Free State, Gauteng, KwaZulu-Natal and Mpumalanga.

### Study population

The population for the interview component was the pharmaceutical services managers within the 11 districts, in South Africa. The main function of the study population was management of essential medicines provision in accordance with the job description of Manager: Pharmaceutical services in the public sector.

The document component was based on the health records from primary healthcare clinics in the 11 districts.

### Sampling

Purposive sampling is used when information rich participants are required, to achieve study objectives (Brink, Van Der Walt & Van Rensburg 2017).

For this study, purposive sampling was used to select the district pharmaceutical services managers as key informants for the study. These managers were selected because they have control over the information and experience of medicine supply in the health districts.

The primary healthcare clinics’ records on placement of post-basic pharmacist assistants and the medicine surveillance reports in the 11 districts were also purposively selected to obtain data on the medicine supply in the primary healthcare clinics.

### Data collection

Data collection in qualitative studies follows a narrative approach (Creswell & Poth 2018). Two data collection tools were developed and utilised in the conduct of this study.

Firstly, a self-designed data extraction tool for completion by the district pharmaceutical services managers was developed and used to collect data on the placement of post-basic pharmacist assistants and medicine availability in each of the primary healthcare clinics within the 11 districts.

Secondly, a self-designed semi-structured interview guide was developed based on the research objectives and the questions related to description of the medicine supply management practices within the primary healthcare clinics to collect data from the interviews with the district pharmaceutical services managers.

The tools were piloted in one district pharmaceutical services management office. Based on the results of the pilot district, which suggested clarity on the category of pharmacist assistant targeted, the data collection tools were refined for use in the formal data collection process. The pilot district was not included in the formal data collection process.

Placement lists of post-basic pharmacist assistants and documents on medicine availability were requested from the 11 district offices. These lists and documents were reviewed to identify the number of primary healthcare clinics with (and without, respectively) appointed post-basic pharmacist assistants and the availability of essential medicines over a period of 12 months.

Semi-structured interviews were conducted with the district pharmaceutical services managers in their offices, with regard to the medicine supply management practices at the primary healthcare clinics. The interviews aimed to obtain participants’ recommendations on how to promote good medicine supply management practices at primary healthcare clinics in the 11 districts. Interviews were audio recorded with the permission of the interviewees.

### Data analysis method

Data analysis is the systematic organisation and synthesis of research data. Qualitative data collected from the document review and the semi-structured interviews can be analysed using Colaizzi’s (1978) seven steps (Polit & Beck 2017).

The seven steps were applied for this study in the following manner: Collected data were read for understanding and to familiarise the researcher with the content. The data files were reviewed to identify and extract significant statements in relation to the study objectives. Meanings were extracted from the experiences of the participants. Data were organised by formulating meanings into clusters of themes and discrepancies between various clusters of data were observed. A detailed description was presented by integrating the results into a detailed description of the participants’ experiences. A fundamental description of the meaning of the experience was produced through identification of essential themes. The results were then shared with the participants for their comments and inputs as a final validating step. The themes and sub-themes are presented under the results section of the article.

### Ethical considerations

Ethical clearance for this study was obtained from UNISA College of Human Sciences Ethics Committee, approval number: 90195108CREC. Gatekeepers’ permission was obtained from the provincial research committee and identified District Managers. Potential participants were provided with an information sheet detailing purpose, process, potential benefits and risks associated with participation in the study. In all, 11 district pharmaceutical services managers provided informed consent for participation in the study. Anonymity was promoted by using pseudonyms to label reported information. Permission to record the interview proceedings was obtained in writing from the participants.

### Trustworthiness

Gray, Grove and Sutherland (2017) proposed that qualitative studies should comply with the principles of trustworthiness including credibility, dependability, confirmability, authenticity and transferability. These principles were adhered to in this study as discussed here.

Credibility was ensured through purposive selection and engagement, over time, of information-rich participants who were qualified pharmacists. Data collected were referred back to the participants for confirmation. Dependability was promoted through the use of standard data collection tools for the two districts. Confirmability was achieved through an audit trail of audiotaped records of the interviews, as well as analysed documents reviewed by independent co-coders. Participants’ quotes were reported as stated during the interviews. Authenticity was ensured by referring back to the participants for their confirmation of the data collected. Transferability was ensured by describing the context in which the study was conducted. The research design, data collection as well as analysis procedures are presented in detail for anyone interested in conducting a similar study – to understand how the study was executed.

## Results

### Demographic profile of the participants

Eleven district pharmaceutical services managers participated in the study: four of them were male and seven were female. The average experience in the medicine supply management was at least 5 years for each of the participants.

### Themes

The following three themes emerged from the study:

1. There is a shortage of post-basic pharmacist assistants in primary healthcare clinics.
2. Post-basic pharmacist assistants’ placement in primary healthcare clinics improves medicine availability.
3. There is a defined role for post-basic pharmacist assistants in the primary healthcare clinics.

#### Theme 1. There is a shortage of post-basic pharmacist assistants in primary healthcare clinics

The study found that 10 of the 11 districts had at least one post-basic pharmacist assistant placed in the quarter of the primary healthcare clinics under their jurisdiction whereas in one remaining district, there were no post-basic pharmacist assistants placed to manage medicine supply in the primary healthcare clinics.

The participants explained the shortage as follows:

‘Not all our clinics have the pharmacist assistants, most of the time medicine supply is carried out by nurses.’ (Pharmaceutical Services Manager 10, female)

‘Pharmacist assistants are very scarce in our district, we are dependent on nurses to manage medicines.’ (Pharmaceutical Service Manager 2, male)

The findings are in line with those of Ogbodu, Maputle and Mabunda (2019), who reported limited availability of pharmacist assistants at primary healthcare clinics. The shortage of pharmacist assistants is a major contributor to essential medicine stockout at these clinics. Matema (2020) also confirmed that although pharmacist assistants are deemed capacitated and skilled to play a key role in medicine supply management at clinics, a shortage of pharmacist assistants still exists, leaving nurses to manage medicine supply at most of the primary healthcare clinics.

#### Theme 2. Post-basic pharmacist assistants’ placement in primary healthcare clinics improves medicine availability

In 10 districts where at least a quarter of their primary healthcare clinics had post-basic pharmacist assistants, the average medicine availability was at an average of 95% – the figure was in line with an acceptable norm of 95%. In one district without post-basic pharmacist assistants, the average medicine availability was an average of 88%.

The district pharmaceutical service managers explained as follows:

‘We are doing well on medicine availability thanks to the availability of (Pharmacist) assistants in our clinics.’ (Pharmaceutical Services Manager 6, female)

‘My district is forever below 95% availability as I don’t have dedicated pharmacist assistants to manage stock and place orders.’ (Pharmaceutical Services Manager 11, male)

Bateman (2015) indicated that causes of stockouts were related to logistical challenges between the depots and primary healthcare clinics at both provincial and district levels. The contributing factors include, among others, a shortage of dedicated pharmaceutical personnel to manage medicine supply at the primary healthcare clinics (Stop Stockout 2018).

#### Theme 3. There is a defined role for post-basic pharmacist assistants in the primary healthcare clinics

The findings of the document analysis were discussed with the pharmaceutical services managers at district level to understand the role of the post-basic pharmacist assistants in the medicine supply chain, with due consideration of the medicine supply chain framework, which focuses on selection, procurement, distribution and rational medicine use. The study found that post-basic pharmacist assistants play a major role in procurement (ordering) and distribution of essential medicines. However, the pharmacist assistants have a minor role in selection and rational medicine use.

**Sub-theme 3.1. Post-basic pharmacist assistants have a limited role to play in the selection of essential medicines:** The pharmaceutical services managers within the districts indicated that selection of essential medicines is a highly technical process that is reserved for the pharmacists in the established structures such as pharmaceutical therapeutic committees; thus, the pharmacist assistant has a limited impact on the selection of essential medicines.

The participants stated:

‘The pharmacist assistants (post basic) are mid-level workers therefore, they have no role in selection of essential medicine.’ (Pharmaceutical Services Manager 1, female)

‘Selection of medicines is beyond the scope of the pharmacist assistants (post basic).’ (Pharmaceutical Services Manager 8, male)

Ziba et al. (2014) agreed that pharmacy certified or qualified candidates improve performance in medicine supply chain management and increase access to medicines at public healthcare facilities. The participants were not of the view that post-basic pharmacist assistants should contribute to the selection of essential medicines as the function is seen as being the responsibility of a registered pharmacist guided by the Pharmaceutical and Therapeutics Committees.

**Sub-theme 3.2. The post-basic pharmacist assistants play a major role in the procurement of essential medicines:** The district pharmaceutical services managers unanimously agreed that post-basic pharmacist assistants have a major impact on the procurement (ordering) of essential medicines because they are specifically prepared to manage medicine supply at primary healthcare clinics.

The participants reported as follows:

‘The availability and provisioning of essential medicines is [sic] positively improved when there is a post-basic pharmacist assistant, because nurses have little interest in medicine stock management as a result oflong patient queues in the clinics.’ (Pharmaceutical Services Manager 4, female)

‘The post-basic pharmacist assistants understand the ordering process for essential medicines, making the medicine ordering process smooth in the clinics.’ (Pharmaceutical Services Manager 6, female)

**Sub-theme 3.3. Post-basic pharmacist assistants play a major role in the distribution of essential medicines:** The National Stop Stock-Out Audit indicated that the causes of stockouts were related to logistical challenges between the depots and primary healthcare clinics, at both provincial and district levels (Bateman 2015).

The district pharmaceutical services managers stated:

‘The pharmacist assistants (post basic) in our district have improved distribution of essential medicines by ensuring proper placement and follow of orders.’ (Pharmaceutical Services Manager 9, female)

‘There is a network created by the pharmacist assistants (post basic) to move medicines between the facilities, which is very helpful in improving availability of medicines in the clinics.’ (Pharmaceutical Services Manager 1, female)

This study confirmed that at primary healthcare clinics where there are post-basic pharmacist assistants, medicine availability was above 90%, which the district pharmaceutical services managers attributed to the placement of the post-basic pharmacist assistants, as the workforce is dedicated to ensure that medicines are distributed, as scheduled, from the depots to the facilities. Furthermore, post-basic pharmacist assistants champion the movement of excess stock between facilities.

**Sub-theme 3.4. Post-basic pharmacist assistants play a limited role in the rational use of essential medicines:** The district pharmaceutical services managers did not consider rational medicine use as an area of independent practice for the post-basic pharmacist assistants. As such, their impact was found to be limited.

The district pharmaceutical services managers expressed their views as follows:

‘Post-basic pharmacist assistants are not independent practitioners, as such may not contribute much to rational medicine use.’ (Pharmaceutical Services Manager 10, female)

‘It is difficult for post-basic pharmacist assistant to make [*sic*] a meaningful role in rational medicines as professional nurses do not view them as peers.’ (Pharmaceutical Services Manager 11, male)

South African Pharmacy Council (2020) supports the district pharmaceutical service managers’ view that the role of post-basic pharmacist assistant comprises that of a mid-level worker, with no direct responsibility for rational medicine use.

## Discussion

The study found that primary healthcare clinics without post-basic pharmacist assistants were more likely to have erratic medicine supply management practices. In addition, the study results confirmed that post-basic pharmacist assistants play a positive role in the medicine supply management processes in primary healthcare clinics.

The findings are supported by various authors including Stop Stockout (2018) who reported that in 2017, primary healthcare clinics across nine provinces have experienced that at least one essential medicine from the basket of vaccines and antiretroviral, tuberculosis was out of stock. The contributing factors included a shortage of dedicated post-basic pharmacist assistants to manage medicine supply at primary healthcare clinics. Bateman (2015) supported the view that the causes of stockouts are related to a shortage of pharmacy personnel, as well as logistic challenges between the depots and primary healthcare clinics at both provincial and district levels. There is a need for each primary healthcare clinic to have at least one post-basic pharmacist assistant to manage medicine supply in order to promote consistent availability of essential medicines.

Mokgathla and Kadama (2017) reported that in the South African primary healthcare clinics, medicine supply management is handled by nurses, who are required to divide their attention between patient care and medicine stock management. Consequently, both functions are compromised. Furthermore, in a study conducted in South Africa, on nurses’ perceptions of the causes of essential medicines stockout, the nurses reported that a shortage and the limited availability of pharmacist assistants are major contributors to essential medicine stockout at the clinics (Ogbodu et al. 2019). Accordingly, the study recommends that medicine stock management at primary healthcare clinics should be handled by post-basic pharmacist assistants.

South Africa can learn from other African countries. Malawi, for example, has identified the medicine supply chain as a priority to overcome key barriers to consistent medicine supply in nurse-driven settings. A new approach was implemented through training and deployment of an enhanced pharmacy assistant cadre. The programme demonstrated that pharmacy certified or qualified candidates improve performance in the medicines supply chain and increase access to medicines at public healthcare facilities (Ziba et al. 2014). The South African National Department of Health, partners and donors have also trained pharmacist assistants to support improvements in medicine supply management across the primary healthcare clinics (Matema 2020). However, the trained pharmacist assistants do not get employed after training. They can be appointed permanently and placed in the clinics to manage medicine supply, thus improving medicine availability and allowing nurses to focus on the clinical care provision.

### Strengths of the study

This study was based on the data collected over a 12-month period with regard to medicine availability and the placement of the pharmacist assistants at primary healthcare clinics, which enabled assessment of trends over a longer period across 685 primary healthcare clinics. Of these clinics, 256 were found to be without a pharmacist assistant employed to manage medicine supply.

### Limitations of the study

The study was conducted based on the data and experiences of district pharmaceutical services managers in 11 out of 52 health districts in South Africa. As such, the findings are not generalisable.

### Study recommendations

The study has demonstrated that post-basic pharmacist assistants are essential in medicine supply management in primary healthcare clinics. South African National Department of Health should allocate funds and strive to appoint at least one post-basic pharmacist assistant at each primary healthcare clinic to improve medicine supply management and medicine availability.

Further studies should be conducted to explore whether the post-basic pharmacist assistants should be capacitated to play an advisory role in rational medicine use – as they are, in most cases, the only pharmacy personnel available at a primary healthcare clinic.

## Conclusion

The data collected through the study revealed that post-basic pharmacist assistants play a positive role in the medicine supply management in primary healthcare clinics. Post-basic pharmacist assistants’ placement at each primary healthcare clinic will contribute towards improving medicine stock management and promoting consistent medicine availability in the primary healthcare clinics.
